# Protein Kinase A in Cancer

**DOI:** 10.3390/cancers3010913

**Published:** 2011-02-28

**Authors:** Antonio Caretta, Carla Mucignat-Caretta

**Affiliations:** Department of Human Anatomy and Physiology, University of Padova, Via Marzolo 3, 35131 Padova, Italy; E-Mail: antonio.caretta@unipr.it

**Keywords:** cancer, cAMP, PKA, diagnosis, therapy

## Abstract

In the past, many chromosomal and genetic alterations have been examined as possible causes of cancer. However, some tumors do not display a clear molecular and/or genetic signature. Therefore, other cellular processes may be involved in carcinogenesis. Genetic alterations of proteins involved in signal transduction have been extensively studied, for example oncogenes, while modifications in intracellular compartmentalization of these molecules, or changes in the expression of unmodified genes have received less attention. Yet, epigenetic modulation of second messenger systems can deeply modify cellular functioning and in the end may cause instability of many processes, including cell mitosis. It is important to understand the functional meaning of modifications in second messenger intracellular pathways and unravel the role of downstream proteins in the initiation and growth of tumors. Within this framework, the cAMP system has been examined. cAMP is a second messenger involved in regulation of a variety of cellular functions. It acts mainly through its binding to cAMP-activated protein kinases (PKA), that were suggested to participate in the onset and progression of various tumors. PKA may represent a biomarker for tumor detection, identification and staging, and may be a potential target for pharmacological treatment of tumors.

## 1. Different Protein Kinases Are Involved in Cancer and Can Be Therapeutically Targeted

Various signal transduction pathways are involved in regulation of cell growth, thus their impairment may be related to tumor pathogenesis. Phosphorylation is the most important reversible mechanism for triggering or inhibiting the activity of specific proteins in a signaling pathway. This fundamental process is achieved through the activity of various protein kinases. The first anticancer agent specifically targeted to a protein kinase was Imanitib, which acts as an inhibitor of the oncogenic kinase BCR-Abl and is active in the chronic myelogenous leukemia [1]. Since then, a feasible therapeutic approach, based on kinase inhibitors, that directly interferes with tumor-specific intracellular signaling pathways, has been a major target in anti-tumor drug design.

## 2. The cAMP-Mediated Signaling Pathway and Its Effectors

The first intracellular second messenger was described in the late Fifties as adenosine 3′5′-cyclic monophosphate (cyclic AMP, cAMP) [2]. It is present in every cell, where it is synthesized by adenylyl cyclase from ATP, and is hydrolyzed by cAMP-specific phosphodiesterases to adenosine 5′-monophosphate. The rate of cAMP production and degradation is sensitive to a wide range of extracellular and intracellular signals, such that cAMP can directly regulate a variety of cell functions, from metabolism to ion channel activation, cell growth and differentiation, gene expression and apoptosis [3]. On the other hand, the cAMP pathway intermingles with other intracellular signaling pathways, from Ca^2+^-mediated [4] to cytokine pathway, via Jak/STAT inhibition [5]. It also strictly interacts with the Ras-mediated MAP kinase, to modulate cell growth events [6].

Within each cell, cAMP may activate different proteins. For example, in olfactory receptors, cAMP may operate directly on ion channels [7]. It may bind to guanine nucleotide exchange factors (Epac: exchange protein directly activated by cAMP), and its downstream effectors may also activate the transcription factor CREB (cAMP-responsive element binding protein). However, its best known mode of action is through binding to cAMP-dependent protein kinases (PKA) [8]. PKA are ubiquitous intracellular cAMP effectors that regulate multiple processes. Their target is specified by their intracellular localization, obtained through anchoring at specific sites in macromolecular complexes, and through the expression of specific subunits. During cancer pathogenesis, the normal cell activity is imbalanced via mutation of selected proteins, or by altering their rate of synthesis/degradation, or by affecting the activity of otherwise normal proteins. Given the involvement of PKA in several different intracellular functions, it is conceivable that pathological processes may affect the cAMP/PKA pathway. Indeed, several converging data reveal that the cAMP/PKA signaling pathway is altered in different cancers, and may be exploited for cancer diagnosis and/or therapy.

PKA are inactive tetramers of two regulatory and two catalytic subunits. When each regulatory subunit binds two molecules of cAMP, the catalytic subunits are released and in turn phosphorylate a variety of target proteins, ultimately modifying their biological activity. There are four regulatory subunits (RIalpha, RIbeta, RIIalpha, RIIbeta) that are differentially expressed in several cells [9]. The general structural features of PKA are retained in all the four regulatory isoforms, with apparently only minor changes in biochemical properties [10]. Three catalytic subunits (Calpha, Cbeta, Cgamma) may be combined to the regulatory subunits to obtain enzymes with different biochemical properties.

During both physiological and pathological conditions, the composition of the PKA holoenzyme as well as their intracellular localization may change, inducing different effects [11].

Noteworthy, in the same cell, elevation of cAMP and subsequent PKA activity by different agonists leads to different physiological responses [12], probably because receptors for extracellular signaling molecules can activate only a fraction of PKA, that are largely segregated in subcellular microdomains by a great number of PKA Anchoring Proteins (AKAPs). AKAPs are bound to cytoskeletal proteins or organelles and bind regulatory subunits of the PKA, so that the PKA can be docked and concentrated close to crucial targets and, despite their broad substrate specificity, can phosphorylate only selected proteins [13,14].

## 3. Variations in PKA Regulatory Subunit Distribution during Development

Most of the above mentioned results have been obtained in cell culture models, however variations in PKA subunits' expression and distribution may characterize different cells also in the living organism. Moreover, variations in PKA expression and distribution may be related to different properties of the same cell type during physiological changes, for example during developmental maturation or ageing, or during pathological modifications.

As an example, we will briefly introduce the variations in differential distribution of the four PKA regulatory subunits inside the brain, both at a regional and subcellular level, with a special focus on the insoluble fraction, bound by cytoskeleton and membrane/organelles [15–18]. In the rodent brain, insoluble RIIbeta is present in most neural and glial cells, while insoluble RIalpha is neuronal and restricted to some brain nuclei. Insoluble RIbeta is present only in some neuron type (olfactory bulb mitral cells, cerebellar Purkinje cells), while insoluble RIIalpha is localized only on the ependymal cells lining the ventricles [19–21]. Each subunit appears at precise times during development and persists for a different time during the lifespan. For example, RIalpha appears postnatally and persists for two months in cerebellar nuclei, while in the archicerebellar granuli it appears from day 17 [20], a period in which Purkinje cells acquire their mature phenotype and express RIbeta [19] suggesting that their expression and intracellular localization is related to the different cell characteristics during the normal developmental process. This distribution is similar in different species [22] but is modified after traumatic or chemical lesions [19,23].

The ontogenetic development of the expression of each subunit and their modification after chemical or surgical lesions, suggested that a different balance between regulatory subunits expression and intracellular localization may be responsible for different properties of the same cell during the normal developmental process. Therefore, it is possible to characterize each cell type, either healthy or not, by different molecular markers, among which we proposed to include PKA subunit expression and their intracellular distribution.

## 4. Involvement of PKA in Cell Cycle Regulation, a Key Event in Cancer Development

Multiple intracellular signaling pathways modulate various events during cell proliferation. cAMP and PKA play different roles in this process [24]. Low cAMP levels are detected at mitosis, while higher levels are present in G1 and early S; on the other hand, PKA phosphorylate macromolecular complexes responsible for the destruction of mitotic cyclins and separation of the sister chromatids at anaphase-metaphase transition [25]. PKA may act sinergistically with Epac to induce mitogenesis in endocrine cells [26].

Different effects may be apparent in different cell lines, since cAMP acts as growth activator in PC12 and Sertoli cells [27,28], while it inhibits growth in NIH3T3 cells and adipocytes [29,30]. cAMP analogs inhibit proliferation and induce differentiation in glioma and neuroblastoma cells [31–33]. These different results may be related to the different expression and/or intracellular distribution of the proteins involved in cAMP cascade.

AKAPs, which anchor PKA at specific sites, are localized at the centrosome [34], whose deregulation has been linked to genome instability and tumor formation [35]. The interactions between AKAPs and RIIalpha are cell-cycle dependent and lead to chromatin remodeling during mitosis [36]. This process is targeted in pathological events, for example the activation of RIIalpha leads to the block of apoptosis during viral attacks [37].

By modulating the timing and localization of cAMP production, it is possible to affect the activation of cAMP effectors, that in turn acts on the RAS/ERK and/or Hedgehog signaling pathways, which are involved in cell cycle progression [38].

## 5. Involvement of PKA Regulatory Subunits in Different Cancers

PKA is involved in the regulation of cell proliferation by acting on transcription factors; for example it may inhibit proliferation by uncoupling Ras from c-Raf activation [39]. The cAMP-mediated pathway is linked to Ras activation through multiple steps, and includes a negative feedback through the phosphorylation of phosphodiesterases (PDE) that ultimately decreases cAMP concentration [39].

The correct PKA cascade is necessary for the functional regulation of D-type cyclins, such that a defective cAMP/PKA pathway may induce carcinogenesis in neuronal precursors [33]. This event may be influenced and even reverted by modifying the type of PKA subunit that is preferentially expressed by the cell [40]. Therefore, the modifications in PKA distribution or activity observed during development or transformation may be relevant to trace cell fate, being eventually targeted to modify the cell phenotype. Different cAMP analogs have been already used to specifically address PKA RI and RII in various diseases [41].

The first link between PKA regulatory subunits and cancer development in human patients was established on the association of RIalpha loss of heterozygosity with endocrine tumors, in the Carney complex [42]. In this disease, the decrease in RIalpha makes more PKA catalytic subunits available, resulting in an enhancement of PKA phosphorylating activity, which ultimately leads to tumorigenesis. Therefore, RIalpha was suggested as a candidate tumor-suppressor gene [43], probably acting on cyclin D1 [44]. Also in lung cancer cells, the increase in PKA type I isozyme induces a nontumorigenic phenotype, while its decrease is followed by acquisition of tumorigenic properties [45]. Deregulation of the effector molecule, cAMP, is involved in cancer genesis [46]. Reduction in cAMP has an anti-proliferative effect on colorectal cancer cells [47]. Cortisol-secreting adrenocortical tumors show a defective expression of RIIbeta, whose stimulation induces apoptosis in the same cells [48]. In prostate carcinoma cells, an increase in RIIbeta expression inhibits tumor growth, while an increase in RIalpha stimulates tumor growth [40]; in these tumors, the cAMP pathway may also interact with the androgen receptor, by enhancing its activation [49].

**Figure 1.**
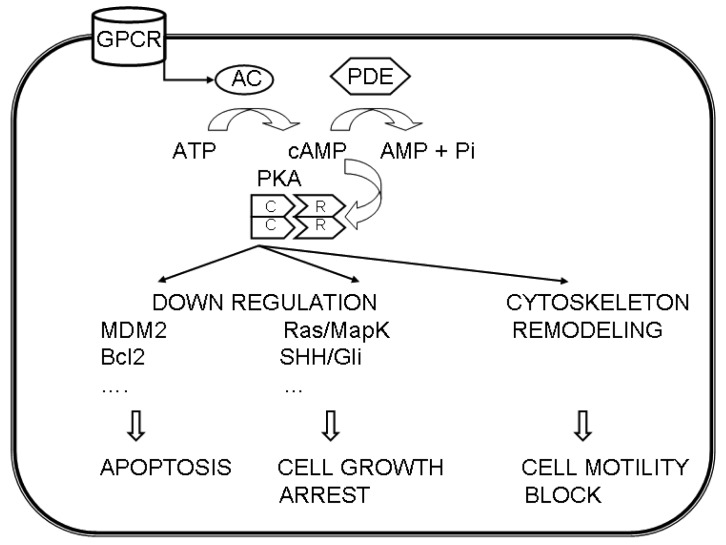
A simplified scheme of the possible involvement of cAMP/PKA pathway in cancer. Adenylyl cyclase (AC) may be activated in several ways, including G-protein-coupled receptors (GPCR), leading to the formation of cAMP, that binds to the regulatory (R) subunits of PKA, thus releasing the catalytic subunits (C). In turn, these phosphorylate several target proteins, leading to apoptosis, arrest of cell growth and block of the cell motility.

These data suggest a different involvement of the PKA regulatory subunits in different cancers via multiple pathways (Figure 1).

The growth of various tumors has been linked to the cAMP/PKA pathway.

The development of Ewing sarcoma requires the involvement of CREB-binding proteins, pointing to a cAMP-mediated pathway [50].

Concerning glioblastoma multiforme, the most aggressive brain tumor, an involvement of protein kinase C is required for tumor growth [51]. However, it has long been known that PKA are ten-times more abundant in GBM than in the normal brain [52], that glioma cells contain PKA type II, and that after cAMP stimulation, the catalytic subunits redistribute to particulate fractions [53].

The activation of cAMP pathway through PKA RII causes differentiation and apoptosis in glioma cells [54]. Noteworthy, AKAP1, a protein that docks PKA to cytoskeleton, was found to be upregulated in glioblastoma specimens, as was phosphodiesterase1A, a cAMP-degradating enzyme [55] while the catalytic subunit of PKA is reduced in high-grade gliomas [56].

Prompted by the above considerations, we screened rodent and human glioblastoma cell lines for the presence and intracellular localization of the four PKA regulatory subunits. At variance with healthy brain tissue, in which RIalpha and RIIbeta are widely present in both soluble and insoluble fraction, these glioblastoma cells present a striking hotspot of insoluble RIIalpha in their Golgi apparatus; in addition, the interference with PKA activity leads these cells to apoptotic death [57] and is possibly correlated with genetic abnormalities on chromosome 7 and 17 [58]. These abnormalities span over the PKA RIalpha, RIbeta and RIIbeta-coding genes: in this way, the balance of PKA RI to RII subunits may be altered and may drive cells to tumorigenic phenotype.

This is consistent with a report on human neuroblastoma cells that shows an increase in RIIalpha after cAMP activation [59]. The growth of neuroblastoma cells is also inhibited by increase in cAMP, which inhibits phosphatidylinositol 3-kinase [60].

Interactions of second messenger pathways controlling cellular functions critical to maintaining cancer characteristics may be reversed by changing intracellular conditions. For example, increasing cAMP concentration may shift activation pattern from PKA RI (high affinity) to RII (lower affinity) subunits [61].

The amount of intracellular cAMP varies during the cell cycle in malignant gliomas, being higher in G0G1 and lower at mitosis: it arrests the cell cycle and induces differentiation and apoptosis, possibly by altering the rate of subunit degradation [54]. On average, glioblastoma cells show a lower cAMP and adenylyl cyclase activity, compared to healthy brain tissue [32].

An increase in the intracellular levels of cAMP, induced by different stimuli, triggers a change in glioma cell morphology and differentiation, while their proliferation is inhibited [62–64]. The effects of PKA on glioma cells are mediated also by the modification of transcription. cAMP-induced differentiation results in the decrease of a number of proteins, including c-jun [65]. On the other hand, the transcription of other proteins is enhanced, for example GFAP [66,67]. Therefore, the activation of PKA in glioma cells induces a number of processes that ultimately lead to differentiation. It has also been suggested that alterations of the cAMP pathway may initiate the immortalization phase of carcinogenesis [66].

Medulloblastoma is a cancer of the cerebellum. During cerebellar development, Purkinje cells secrete Sonic Hedgehog factor, that induces granule cell precursors proliferation: this effect is inhibited by adenylyl cyclase activation [68]. Medulloblastoma cells, similarly to previous findings, decrease their growth rate and start differentiation after increasing cAMP [69,70]. PKA activity is essential to prevent the expression of Hedgehog effectors like the protein GLI [71], since activation of G-protein coupled receptors induce PKA activation, which represses GLI activity [72]. Apparently, PKA downregulates Hedgehog signaling through different mechanisms, by promoting both proteolysis and GLI interaction with other proteins [73]. In the developing cerebellum, adenylyl cyclase activity is inhibited by the activation of the chemokine Gi-coupled receptor CXCR4 [74]. When CXCR4 activity is blocked, and hence cAMP production increases, medulloblastoma growth is inhibited, similarly to phosphodiesterase blockade [75]. A differential distribution of PKA regulatory subunits is typical of medulloblastoma [76], and may be related to PKA role in medulloblastoma pathogenesis.

Another function, in which PKA may operate and may be dysregulated in cancer, is the actin-based cell migration, that involves cytoskeleton remodeling. PKA regulates actin dynamics, by targeting structural proteins, like actin, integrins, VASP and myosin light chain, and regulatory proteins, like Rho GTPases, Src kinases, p21-activated kinases, phospatases and proteases [77]. The involvement of PKA in migration of breast carcinoma cells has been described [78].

## 6. PKA as a Potential Target for Tumor Therapy

Several commercial drugs and peptides (for exampe, forskolin, CREBtide, KEMPtide, PKA RIIalpha autophosphorylation peptide, the myristoylated inhibitory peptide PKI 14-22amide) or chemical treatments (zinc sulfate) are known to interfere with cAMP synthesis or degradation, or to activate or inhibit PKA activity [40]. Type I and type II regulatory subunits of PKA can bind with a relative selectivity 8-substituted and 6-substituted molecules of cAMP, respectively. Some of these cAMP analogs have also been tested as anticancer agents, aiming for selective stimulation of PKA RI [79–81]. PKA activity may also be interfered with by targeting the catalytic subunit, since it may act also as a scaffold to allow diverse interactions with various proteins [10]. In addition, phosphodiesterase inhibitors have been used as potential anti-cancer drugs in combination with conventional chemotherapy [82,83].

A potential role for PKA targeting has been proposed for lung cancer treatment, due to its involvement in acetylcholine receptor signaling [84]. Activation of type II PKA may have an anti-leukemic effect in a rat model of acute myeloid leukemia [85]. Recently, it was shown that in different cancer cell lines, the PKA-induced activation of NF-kappa-B is determined by accessory proteins like the A-kinase-interacting protein 1, so that the effect of PKA inhibition for anti-cancer therapy can be precisely predicted [86].

An aspect different from kinase activation is the manipulation of kinase expression. This is particularly intriguing, because of compensatory increase of one PKA regulatory isoform when a different isoform's transcription is suppressed [40]. Some PKA antisense oligonucleotides [87,88] have already been used to block RIalpha expression in tumors in which RIalpha seems to be involved, with subsequent antitumor activity.

## 7. PKA as an Aid for Tumor Diagnosis

The dysregulation of PKA signaling in several types of cancer suggested abnormal PKA presence in patients should be investigated. In human endocrine tumors, an imbalance in RI/RII ratio has been detected [89]. In the multiple tumors of the Carney complex, different mutations in the RIalpha have been detected, that induce a lower synthesis of the normal protein, resulting in increased PKA catalytic subunit activation, so that the altered or missing expression of PKA RIalpha may be a marker for this disease [90]. On the other hand, RIalpha overexpression has been successfully used also as a predictor for prostate cancer outcome [91,92].

The overexpression of PKA is a trait common to various types of cancer. Part of the PKA that is synthesized by a cancer cell is secreted, and is found indeed as extracellular PKA in the serum of cancer patients. A method based on PKA autoantibody detection revealed that cancer patients can be reliably discriminated from controls [93]. In addition, the PKA extracellular activity is consistently higher in cancer patients [94]. These data support the role of PKA as a general marker for various types of cancer.

In conclusion, recent increasing evidence points to PKA as a viable tool for tumor diagnosis and suggests PKA as a potential target for tumor therapy.
